# The transition from learner to provider/teacher: The learning needs of new orthopaedic consultants

**DOI:** 10.1186/1472-6920-5-17

**Published:** 2005-05-17

**Authors:** Brian McKinstry, Malcolm Macnicol, Katy Elliot, Stuart Macpherson

**Affiliations:** 1 General Practice Division, Community Health Sciences, University of Edinburgh, 20 West Richmond St., Edinburgh UK; 2 Department of Orthopaedics, Royal hospital for Sick Children, Sciennes Road, Edinburgh, UK; 3 NHS Education, Lister Institute, 11, Hill Square Edinburgh UK

## Abstract

**Background:**

Given the relatively sudden change from learner to teacher-provider that new consultants experience and the likely clinical and managerial challenges this may pose, there is a relative dearth of research into the problems they may have in relation to their new roles, or how supported they feel by senior colleagues acting in a mentoring role. This research sought to determine new consultants views on the quality and relevance of their training, its relationship to their confidence in clinical and managerial skills and their views on mentorship by senior colleagues.

**Methods:**

Detailed postal questionnaire to new consultants using open and closed questions.

Open questionnaire to established consultants to validate new consultant responses.

**Results:**

Respondents felt their clinical training was good and were generally confident in most clinical skills although some perceived deficiencies in more complex procedures and specialist areas. Most lacked confidence in many managerial skills. These perceptions were verified by established consultants. Although no relationship was found between total training time or quality of training with confidence, extra training in specific sub-specialities improved confidence in these areas. While most established consultants thought that mentorship would be useful for new consultants, only 52% of them shared this view.

**Conclusion:**

Training and experience in management should be given greater emphasis. There may be a need for specific, targeted training in complex procedures for doctors who experience lack of confidence in these areas. Mentorship should be offered to new consultants and recognised in the job-plan of the new consultant contract.

## Background

Every year in the UK over a thousand doctors move from training grades to consultant posts. Educational research tends to focus on training grades and there is a relative dearth of information on the problems these new consultants have in relation to their new roles particularly their perceived learning needs in relation to clinical and management issues. Considering the relatively sudden change from learner to teacher-provider and the likely challenges this may pose, it is not clear to what extent new consultants in their early years feel supported by senior colleagues acting in a mentor or teaching role to aid them with this transition. The recent shortening of training for UK consultant posts following the Calman report [1] in the mid 1990s and the reduction in working hours during training leading up to the full implementation of the European Working Time Directive have led to concerns that new consultants, particularly surgeons, may be less well prepared than their predecessors to face the challenges of their new role [2,3].

Against this background we chose to investigate the problems new consultants may face. We chose to use orthopaedic surgery as a model for the craft specialties because it is considered to have a consistent training program with a relatively unspecialised end-point. It is also one of the best organised surgical specialties, with a careful, annual census and lastly has a significant number of new consultants appointed annually.

National Health Service Education for Scotland (NES) collaborated with the British Orthopaedic Association (BOA) to survey consultants registered with the BOA who had been in post for less than five years. We sought to determine their self-perceived learning needs and how these were related to their training experience. Their views were sought on the quality and relevance of their training experience, the degree to which they felt supported by senior colleagues in their new posts and their views on formal mentorship. In an attempt to triangulate the new consultant responses on self-perceived learning needs, questionnaires were sent to a small number of established consultants for their views on the problems faced by new consultants.

### Aims

To determine:

• the self-perceived learning needs of new orthopaedic consultants

• if there is a correlation between perceived length and supervision of training or experience abroad and subsequent self-perceived competence

• whether new consultants feel they were given adequate support from senior colleagues in the early years of consultantship and their attitude to formal mentoring

• the views of new consultants and established consultants on the length and quality of current training

• what changes these doctors believe should be made to orthopaedic training

## Methods

### Designing the questionnaires

The content of the questionnaires was decided after a series of face-to-face interviews with specialist registrar surgeons within one year of the end of training (SpR) and established and recently appointed consultants. These surgeons showed a preference for a detailed check list questionnaire which allowed space for free text. The senior consultants interviewed were very clear that for them a short more open questionnaire would be preferred. As the questions to new consultants included the sensitive areas of self-assessed competence interviewees stressed that responses should be anonymous.

The survey for new consultants (see additional file 1) covered the following areas:

• extent and perceived quality of their training

• content and quality of time spent abroad

• access to courses and advice about choice

• perceived competency in the main areas of elective and trauma surgery, managerial and communication skills, teaching and research skills

• the availability of advice and help including attitudes to formal mentoring

• perceived learning/developmental needs

• perception of how well training has prepared them for consultantship

• their main learning needs and when these might have been best addressed

The survey of established consultants (see additional file 2) was an open unstructured questionnaire which asked;

• What they thought were the main problems faced by new consultants

• Their views on the current breadth and depth of training

• How they thought training might be improved

• Their views on the support readily available for new consultants

• Their views on the utility of formal mentoring to new consultants

The survey was piloted on a group of ten new consultants and SpRs. They found the questionnaire relatively straightforward and easy to complete.

## Distributing the questionnaire

Using the database of the BOA the questionnaire was sent to a randomly selected group of 150 consultants who had taken up their first consultant post in the last five years. The decision to use the 5-year cut off was pragmatic in that the BOA has a separate register of this group. In addition we sent the established consultant questionnaire to 100 randomly chosen consultants of greater than five years standing selected from the BOA database. One reminder was sent.

### Analysis

The new consultant questionnaire responses were entered into SPSS. Results were analysed using non-parametric tests (Mann-Whitney or Chi-Square as appropriate). The established consultant responses from open questionnaire were analysed for themes by BM and checked by KE independently.

## Results

### Response rates

90/150 (60%) 'new' consultants and 30/100 (30%) 'established' consultants replied. Questionnaires were generally very well completed with frequent comments made.

### New consultants training experience

The majority of this group graduated between the years 1986 and 1990 (range 1978–1990). Most graduated in the UK (67%), spent 4 years (range 1–7) as a Senior House Officer (SHO) (a junior training grade in the UK), and 6 years (range 3.5–12) as Specialist Registrar (SpR) (a senior training grade in the UK established in the mid 1990s). The majority of these were spent in 'mixed' orthopaedic/trauma posts (58%), with smaller numbers in specialist adult elective orthopaedics (16%), specialist trauma (11%), paediatrics (10%) and research (10%).

### Length of training and quality of supervision

Consultants generally thought that the amount of time gaining experience was 'just right'. A very small number (4.4%) thought that there had been too little time in part of their training. Most consultants thought they had received good supervision in their posts although 18% said they 'could have done with more' and 15% described their supervision as 'often insufficient'. None described it as 'grossly insufficient'.

### Time spent abroad

59 (67%) of the consultants had spent time abroad during their training. The most common destinations were Australia (22), followed by the USA (12), Ireland (7) and Canada (6). Most spent between 6 and 18 months abroad. The majority (77%) spent their time in practical tasks such as operating; 20% mainly observed; and 4% did some research. All but one described the experience as very positive. However we found no relationship between having experienced time training abroad and current confidence in any clinical, teaching or managerial skill.

### Guidance on and availability courses

SpRs in the UK are encouraged to undertake thirty study days a year most of which is spent on organised courses run by the Royal Colleges of Surgeons, BOA or similar organisations. The courses are very varied including sub-specialist subjects such as hand surgery or management topics such as appraisal.

New consultants were unhappy with the guidance they had been given during training with regard to what type of postgraduate courses available both in the UK and abroad would be most appropriate for them to attend. Particularly some felt that advice on which point in their career path to attend a particular type of course would have been useful. Most described the advice as poor or non-existent (73%). Although the majority (72%) felt that they were able to attend courses that were of value to their future career, a sizeable proportion were not, mainly due to either lack of time (19%) or finance (12%). Several commented that courses should be more clearly targeted for specific groups at different stages of training.

When asked; '*Did your clinical training prepare you adequately for your current post?' *77 (90%) concurred. Almost all additional comments rated the clinical training as good.

### How do new consultants rate their skills?

Consultants generally were confident in most practical procedures (table 1) with a few exceptions (largely in specialist areas) in which a proportion reported that they were 'not very' or 'not at all' competent (spinal surgery (79%), paediatric surgery (56%), shoulder surgery (46%), sports medicine (36%) and hand surgery 48%)). However, there was clear lack of perceived skill in a wide range of management areas (see table 2) with half or more of all respondents reporting they were 'not very ' or 'not at all' competent in negotiation, appraisal, business planning, medicolegal skills and managing private practice and, perhaps less importantly for most consultants, some research skills. However most regarded their teaching skills (table 3) and all regarded their communication skills as very or quite good.

**Table 1:** How surgeons responded to the question; 'How would rate your competence in the following skills?'

	***Degree of competence (valid%)***				
***Skill***	***Very***	***Quite***	***Not very***	***Not at all***	***missing***

***Arthroplasties***					
*Knee*	48 (55)	37 (42)	3 (3)	0 (0)	2
*Hip*	45 (51)	43 (49)	0 (0)	0 (0)	2
***Other elective procedures***					
**Knee**	34 (42)	43 (53)	3 (3)	1 (1)	9
*Hip*	25 (32)	45 (57)	7 (9)	1 (1)	12
*Shoulder*	10 (12)	37 (43)	35 (41)	4 (5)	4
*Spinal*	2 (22)	16 (19)	40 (48)	26 (31)	6
*Hand*	15 (7)	53 (27)	17 (48)	0 (0)	5
*Amputation*	15 (17)	48 (56)	19 (22)	4 (5)	4
*Arthroscopy*	49 (54)	37 (41)	2 (2)	0 (0)	2
*Sports medicine*	21 (24)	34 (40)	28 (33)	3 (3)	1
**Trauma**	58 (66)	29 (33)	1 (1)	0 (0)	2
***Paediatric***	11 (13)	26 (30)	44 (51)	5 (51)	4

**Table 2:** How surgeons responded to the question; 'How would rate your competence in the following skills?'

***n = 90***	***Perceived degree of competence (%)***				
***Skill***	***Very***	***Quite***	***Not very***	***Not at all***	***missing***

**Management**					
*Negotiation*	7 (8)	37 (42)	38 (43)	6 (7)	2
*Business planning*	2(2)	17(19)	50 (56)	20 (22)	1
*Financial skills*	3 (3)	14 (16)	48 (54)	24 (27)	1
*Leadership*	20 (23)	54 (60)	12 (14)	3 (3)	1
*Appraisal*	6 (7)	24 (27)	42 (48)	16 (18)	2
*Presentations*	37 (42)	46 (52)	5 (6)	1 (1)	1
*Medico-legal (court work, reports)*	8 (9)	28 (32)	36 (40)	17 (19)	1
*Risk management*	3 (3)	33 (38)	41 (47)	11 (13)	2
*Managing private practice*	4 (5)	14 (16)	39 (44)	32 (36)	1
***Research Skills***					
*Applying for grants*	6 (7)	18 (21)	33 (38)	31 (35)	2
*Running research projects*	10 (11)	33 (37)	29 (33)	17 (19)	1
*Literature review*	26 (29)	51 (57)	8 (9)	4 (5)	1
*Writing up*	23 (26)	40 (45)	19 (21)	7 (8)	1

**Table 3:** How surgeons responded to the question; 'How would rate your competence in the following skills?'

***n = 90***	***Perceived degree of competence (%)***				
***Skill***	***Very***	***Quite***	***Not very***	***Not at all***	***missing***

**Teaching skills**					
*Small group teaching*	47 (53)	39 (44)	3 (3)	0 (0)	1
*Mentoring skills*	18 (20)	58 (65)	11 (12)	2 (2)	1
*Appraising students*	18 (20)	49(55)	20 (23)	2 (2)	1

Neither the total time spent in training nor the perceived supervision and guidance in training was related to self-rated competence. However, those who had spent longer (>12 months) in a sub-specialist area such as paediatric orthopaedics were more likely to perceive themselves as very or quite skilled in that area (16/18 (89%) v 11/19 (58%) p < 0.05). Those who had reported more difficulty in attending 'suitable' courses showed a trend significant at the p < 0.05 level for less confidence in arthroscopy and sports medicine. Those doctors (n = 9) who felt unprepared for consultantship after their training showed a trend for scoring their confidence lower across most clinical skills. They were significantly more likely to describe their communication skills with colleagues as not very good or poor (4/9(44%) v 1/76(1%) p < 0.001).

When asked what were their main learning needs at the moment, the commonest response was in the area of management skills, particularly negotiation skills. Many commented that they had not been at all prepared for the amount of administration and negotiation they encountered as a new consultant. The clinical skills identified as learning needs were most frequently revision arthroplasty (10% of all respondents), followed by spinal surgery, shoulder arthroscopy, foot, ankle and hand surgery. New consultants thought these management skills and specialised clinical learning needs should be best addressed in late SpR training or during fellowship years.

### Availability of support when appointed as a new consultant

Most new consultants (86%) thought that help was readily available when they took up their posts although 44% did have concerns regarding this when they first became a consultant. Some suggested that such help needed to be more structured and that consultants themselves needed to recognise that they should occasionally ask for help. When asked if they would have liked some form of mentoring from a senior colleague when they began working as a consultant, 52% replied that they would have. Others felt that mentoring might be like '*being a Senior Registrar again' *or that some senior colleagues were *'too out of date to be of help'*. A small number was concerned that it might diminish them in front of senior colleagues. Many felt that the support they received informally meant mentoring was unnecessary.

### The views of established consultants

Established consultants (EC) confirmed that, while generally well trained clinically, new consultants lacked managerial and administrative skills. They particularly pin-pointed the importance of negotiation skills, for problems as diverse as getting an office, negotiating suitable theatre and out-patient clinic time with powerful senior colleagues, or negotiating a balance between teaching, private practice and their National Health Service (NHS) clinical commitments:-

"Suddenly thrust into having to negotiate for all they want or need to carry out their plans with no previous experience of management" (EC)

"The new consultants who, in my experience, have shown greatest stress and difficulty have been those who over-stretched themselves with non-NHS work. Sadly only the NHS is blamed for the stress and is expected to provide the solution."(EC)

Established consultants drew a distinction between training which they generally thought good (although occasionally too specialised) and experience, which they were concerned new consultants lacked. Like the new consultants they identified complex clinical problems (e.g. revision arthroplasty) and rare problems as difficult areas. In these cases advice from more experienced surgeons was seen as important:-

"Although many consultants may be knowledgeable in the general sense, they have little experiential knowledge and little practical sense. This is mainly due to reduction in training time, and, in addition, reduction in hours of training."(EC)

"They are expected (in DGHs) to be masters of all specialties within orthopaedics, and generally their training will be deficient in one or more sub-specialties."(EC)

Like the new consultants, experienced consultants felt that more time should be spent on management training in the final years of a program:-

"Introduce training on time management, paper management and teaching techniques."

"5^th ^and 6^th ^year SpRs should have to attend directorate meetings to give them a taste of what is to come." (EC)

Established consultants, however, felt that training needed to be longer than currently, some suggesting clinical fellowships, junior consultant grades and proleptic appointments in addition to current training.

In terms of support, most felt that generally this was good for new consultants. Several perceived that a bigger problem was that of the new consultant who doesn't seek help when it is required:-

"Yes. Those that know to ask for help and seek it are not the problem. Those that don't ask are." (EC)

But two commented:-

"No, it is very rare that newly appointed consultants have access. Even if there is access one tends to be hesitant. We should give them a lot of support, guidance and make them feel very comfortable so they can come forward and ask for help." (EC)

".....*there is a requirement that a 'no blame' culture exists for this to happen fairly, and without fear or favour. Unfortunately, this is not always the case." (EC)*

All but one established consultant thought that some form of mentoring was desirable. Many thought that it happened informally already. Again it was suggested that proleptic appointments could be the basis of a mentored transition to independent practice. Some saw appraisal by a senior colleague as fulfilling this role. One saw advantages to both established and new consultants in this process. There were some caveats, however:

"Yes. Mentors would have to be carefully chosen. A consultant in the same subspecialty is best placed to give advice on difficult clinical cases, but can be less than helpful because he sees a rival and wants to defend his own territory" (EC)

"...better to encourage support of all colleagues" (EC)

## Discussion

A 60% return, while good for a postal questionnaire, begs the question as to how representative the results may be. In particular although well completed and containing much valuable information, the 30% return from the established consultants must be interpreted with particular caution as they may represent the more attentive and professional element. However the main purpose of this part of the survey was to provide some validation of the self perceived skills of the new consultants and generally this was provided by the replies we received.

Several studies suggest that self-assessment of skills is not often corroborated by external observation [4]. There is, however, evidence that highly detailed competencies such as we employed, are more likely to be more accurately self-rated than global skills [4,5]. Still, some results (for example 100% describing their confidence in their patient communication skills as very or quite good when there is general evidence to the contrary [6]) raise concerns about the validity of some of these self-assessments. The results should therefore be viewed with some caution. There are undoubtedly more effective ways of assessing the practice of clinical and managerial skills such as audit, 360 degree assessment or perhaps even ethnographic studies. It may be that with the introduction of regular appraisal and audit in the UK such data may become available for future studies.

While new orthopaedic consultants generally feel their clinical training has been good (apart from poor guidance on suitable courses), the transition from teacher to learner/provider clearly presents them with new challenges for which they feel they are not well prepared. This has also been described by others in the UK and abroad [7-9]. These challenges are largely in the area of management although, understandably, complex procedures such as revision arthroplasty represent another area of concern. New consultants were clear that they required additional training in management and some clinical areas, but both they felt that the timing of this was important. They felt training should occur immediately prior to becoming a consultant or shortly afterwards when it was most relevant to them. They also felt that some involvement in management issues near the end of their training period would be helpful.

These observations were largely confirmed by their more experienced colleagues. While established consultants felt that more time was needed in training, this was not the perception of the newer consultants who believed that targeted training and actual experience (for example attending management meetings) within the current training time could address these areas.

The small number of new consultants who reported that they felt unprepared for their role were much more likely to report that their communication with other colleagues was poor. Interpersonal skills have been described as vital for success as a new consultant [8]. This is an area which requires further research to determine if this relationship is causal and if it has the potential for intervention during training.

Although most had felt they had been well supported informally a majority of new consultants and senior consultants were in favour of formal mentorship in the early years of posts, some were very negatively disposed towards the concept. The introduction of consultant appraisal may help in this area although it will not help with the day-to-day problems faced by new consultants. Where mentorship has been made available it has been found to be helpful [8]. Possibly the best approach is to offer and promote mentorship to those who wish it.

Internationally, health care systems are trying to come to grips with reduced working hours for doctors in training [10-12]. There is *some *reassurance in these results that shortened training has not resulted in serious *self-perceived *skill deficiencies and this is in keeping with a study of trainee satisfaction with training after the Calman reductions in training time [13]. However, it will be interesting to repeat this survey in the future to see if the further reduction in working time brought about by the European Working Time Directive has the consequences so many fear [14].

While this study focused on orthopaedic surgery, there is some evidence that the generic management skills, identified as being problematical, challenge consultants in other specialties [15]. Clearly this is an area which needs to be addressed in training.

## Conclusion

Apart from some concerns about complex and specialist procedures new orthopaedic consultants are generally confident in their clinical competence. However, they perceive important learning needs in management skills. Senior colleagues corroborated these observations. New consultants were generally satisfied with their training but some felt supervision was less than adequate at times and that they had insufficient advice and finacial support for instructional courses. Training in management skills, medicolegal matters and experience of management procedures during training should be given greater emphasis during later SpR years. There may be a need for specific, targeted training in complex orthopaedic procedures for doctors who identify lack of confidence in these areas. Mentorship by senior colleagues should be offered to new consultants, but in a way that is neither prescriptive or demeaning.

## Competing interests

The author(s) declare that they have no competing interests.

## Authors' contributions

BMcK designed the study, carried out preliminary interviews, analysed the data and wrote the paper. MM helped design the study and the questionnaires and helped write the paper. Katy Elliot dispatched the questionnaires and entered the data. SM helped design the study and contributed to the writing of the paper.

## Pre-publication history

The pre-publication history for this paper can be accessed here:



## Supplementary Material

Additional File 1 New consultants' questionnaire. Click here for file

Additional File 2 Established consultants' questionnaire. Click here for file

## References

[B1] Department of Health (1995). A guide to specialist registrar training.

[B2] Bates T (1996). Curricular training and the new deal. Ann R Coll Surg Engl.

[B3] Skidmore D (1997). Junior surgeons are becoming deskilled as a result of Calman proposals. BMJ.

[B4] Gordon M (1991). A review of the validity and accuracy of self assessments in health professions training. Academic Medicine.

[B5] McKinstry B, Peacock H, Blaney D (2004). Can trainers accurately assess their training skills using a detailed checklist? A questionnaire-based comparison of trainer self-assessment and registrar assessment of trainers' learning needs. Education for Primary Care.

[B6] Cartwright A, Windsor J (1992). Outpatients and their Doctors: a Study of Patients Potential Patients, General Practitioners and Hospital Doctors.

[B7] Johnson G, Brown R, Howell M (1997). Higher specialist training in accident and emergency medicine – Past, present and future. Journal Of Accident & Emergency Medicine.

[B8] Houghton A, Peters T, Bolton J (2002). What do new consultants have to say?. BMJ.

[B9] Itani KM, Liscum K, Brunicardi FC (2004). Physician leadership is a new mandate in surgical training. American Journal of Surgery.

[B10] Sprangers F (2002). The Dutch experience of implementing the European Working Time Directive. BMJ.

[B11] Watson PY (2002). The working and learning environment for physicians in training in the US. BMJ USA.

[B12] Warshaw A, Sarr M (2003). The now and future world of restricted work hours for surgeons. Surgery.

[B13] Paice E, Aitken M, Cowan G, Heard S (2000). Trainee satisfaction before and after the Calman reforms of specialist training: questionnaire survey. BMJ.

[B14] Chikwe J, de Souza AC, Pepper JR (2004). No time to train surgeons. BMJ.

[B15] Johnson G, Brown R, Howell M (1997). Higher specialist training in accident and emergency medicine – past, present and future. J Accid Emerg Med.

