# Supplementary material for: The transition from learner to provider/teacher: The learning needs of new orthopaedic consultants
Source: BMC Med Educ. 2005 May 17;5:17. doi: 10.1186/1472-6920-5-17 (PMC1156900; doi:10.1186/1472-6920-5-17)
Supplement: Additional File 1 — New consultants' questionnaire. [file 1472-6920-5-17-S1.doc]

Some questions about your experience and training

| **Medical School** | What year did you qualify? |  | |
| --- | --- | --- | --- |
|  | What university? |  | |
|  |  |  |  |
| ***SHO Experience*** | For how many years were you an SHO? |  |  |
|  | How much time (months) was spent on Orthopaedic specialties? | TOTAL |  |
|  |  | Elective only |  |
|  |  | Trauma only |  |
|  |  | Elective & Trauma Mixed |  |
|  | How much time (months) did you spend in the following related specialties? | General Surgery |  |
|  |  | Accident & Emergency |  |
|  |  | Plastic Surgery |  |
|  |  | Vascular Surgery |  |
|  |  | Neurosurgery |  |
|  |  | Paediatric Surgery |  |
|  |  | Research |  |
|  |  | Other (please state) |  |
|  |  |  |  |
| **SpR** **Experience** | How much time in total (months) did you spend as a registrar (SpR)? |  |  |
|  | How much time (months) did you spend within the following? | Elective |  |
|  |  | Trauma |  |
|  |  | Elective & Trauma Mixed |  |
|  |  | Pure Research |  |
|  |  | Paediatric Orthopaedics |  |
|  | With regard to time spent in individual posts, was it  (Please tick one) | Too much | O |
|  |  | About right | O |
|  |  | Too short | O |
|  | How well do you feel you were supervised during the posts? (Please tick one). Please add a comment if you were not well supervised outlining the type of difficulties you experienced | Too much(would have preferred to get on with things myself) | O |
|  |  | Very well (just about right) | O |
|  |  | Good (would have preferred more) | O |
|  |  | Often insufficient | O |
|  |  | Grossly insufficient | O |
|  | If so were left unsupervised, did this occur  (Please tick one) | Mainly by day | O |
|  |  | Mainly by night | O |
|  |  | Both | O |
|  | Comments: | | |
|  | | | |
| ***Time Abroad*** | Did you spend time abroad? | Yes O No O | |
|  | If so, for how many months? |  | |
|  | Where did you visit? |  | |
|  | What did you mostly do? (Please tick one) | Operating | O |
|  |  | Observing | O |
|  |  | Research | O |
|  | In what terms was your time abroad useful? | Education | O |
|  |  | Personal development | O |
|  | Comments: | | |

| ***Training/Courses*** | In your SpR years how was the guidance given to you regarding which external courses you should attend? (Please tick one) | Good | O |
| --- | --- | --- | --- |
|  |  | Poor | O |
|  |  | Non-existent | O |
|  | Were you able to attend courses the course you thought were important for your future career? | Yes | O |
|  |  | No | O |
|  | If not, what were the reasons (indicate more than one if applicable) | Lack of finance | O |
|  |  | Lack of time (service commitment) | O |
|  |  | Not felt suitable by trainer | O |
|  |  | Not felt suitable by Dean’s office | O |
|  | Comments: | | |

| **Competency** | Try to remember what your skills were like when you first became a consultant. How confident were you in each of these skills?  (Please tick one in each skill) | TECHNICAL/CLINICAL SKILLS | | | | |
| --- | --- | --- | --- | --- | --- | --- |
|  |  | *Elective arthroplasty* | very | quite | not very | not at all |
|  |  | knee | O | O | O | O |
|  |  | hip | O | O | O | O |
|  |  | *Elective (other)* |  |  |  |  |
|  |  | knee | O | O | O | O |
|  |  | hip | O | O | O | O |
|  |  | shoulder | O | O | O | O |
|  |  | spinal | O | O | O | O |
|  |  | hands | O | O | O | O |
|  |  | amputation | O | O | O | O |
|  |  | *Arthroscopy* | O | O | O | O |
|  |  | *Sports medicine* | O | O | O | O |
|  |  | *Trauma* | O | O | O | O |
|  |  | *Paediatric* | O | O | O | O |
|  |  | *Investigative skills* |  |  |  |  |
|  |  | COMMUNICATION SKILLS | | | | |
|  |  | with Patients | O | O | O | O |
|  |  | with Colleagues | O | O | O | O |
|  |  | MANAGERIAL SKILLS | | | | |
|  |  | Negotiation | O | O | O | O |
|  |  | Business planning | O | O | O | O |
|  |  | Financial skills | O | O | O | O |
|  |  | Leadership | O | O | O | O |
|  |  | Appraisal | O | O | O | O |
|  |  | Presentations | O | O | O | O |
|  |  | Medico-legal (court work, reports) | O | O | O | O |
|  |  | Risk management | O | O | O | O |
|  |  | Managing private practice | O | O | O | O |
|  |  | RESEARCH SKILLS | | | | |
|  |  | Applying for grants | O | O | O | O |
|  |  | Running research projects | O | O | O | O |
|  |  | Literature review | O | O | O | O |
|  |  | Writing up | O | O | O | O |
|  |  | TEACHING SKILLS | | | | |
|  |  | Small group teaching | O | O | O | O |
|  |  | Mentoring skills | O | O | O | O |
|  |  | Appraising students | O | O | O | O |

| **Asking for Help** | At present, do you find help readily available when you have a problem? | Yes | O |
| --- | --- | --- | --- |
|  |  | No | O |
|  | Comments : | | |
|  | Did you have concerns regarding this when you become a consultant? | Yes | O |
|  |  | No | O |
|  | Comments : | | |
|  | Would you have liked some form of mentoring from a senior colleague when you began working as a consultant? | Yes | O |
|  |  | No | O |
|  | Comments : | | |

| ***What are your main learning needs at the moment. Please say how they can be addressed and at what stage they could have been or should be addressed*** | Area for development | How should this be addressed (e.g.course, on the job experience) | When is the ideal time for this type of learning need to be addressed |
| --- | --- | --- | --- |
|  |  |  |
|  |  |  |
|  |  |  |

| **Did your clinical training prepare you adequately for your current post?** | **Yes O** | **No O** |
| --- | --- | --- |
| **Comment** | | |
|  | | |
| **What were your main concerns about taking up a consultant post? What have you found most difficult?** | | |
|  | | |
