# Supplementary material for: The transition from learner to provider/teacher: The learning needs of new orthopaedic consultants
Source: BMC Med Educ. 2005 May 17;5:17. doi: 10.1186/1472-6920-5-17 (PMC1156900; doi:10.1186/1472-6920-5-17)
Supplement: Additional File 2 — Established consultants' questionnaire. [file 1472-6920-5-17-S2.doc]

What do you think are the main problems new consultants face when they start work?

Do you feel that current training has sufficient breadth and depth? Please comment.

In what ways could training be improved to address the problems mentioned above?

Do you think new consultants have easy access to advice (on clinical and non-clinical issues from senior colleagues? YES NO

***Comments***

Do you think some form of mentoring from an experienced consultant in the early years of consultantship would be helpful? YES NO

***Comments***
